# The medial condylar wall is a reliable landmark to kinematically align the femoral component in medial UKA: an in-silico study

**DOI:** 10.1007/s00167-021-06683-9

**Published:** 2021-08-07

**Authors:** Benjamin Preston, Simon Harris, Loic Villet, Collin Mattathil, Justin Cobb, Charles Rivière

**Affiliations:** 1 Imperial College London School of Medicine, South Kensington Campus, London, SW7 2DD UK; 2 MSK Lab-Imperial College London, White City Campus, London, W12 0BZ UK; 3 The Lister Hospital, Chelsea Bridge Rd, London, SW1W 8RH UK; 4 Clinique de Sport Bordeaux Mérignac, 04 Rue Georges Negrevergne, 33700 Mérignac, France; 5 Personalized Arthroplasty Society, Montreal, Canada

## Abstract

**Purpose:**

Kinematic alignment (KA) aligns the femoral implant perpendicular to the cylindrical axis in the frontal and axial plane. Identification of the kinematic axes when using the mini-invasive sub-quadricipital approach is challenging in unicompartmental knee arthroplasty (UKA). This study aims to assess if the orientation of condylar walls may be suitable for use as an anatomical landmark to kinematically align the femoral component in medial UKA. It was hypothesised that the medial wall of the medial condyle would prove to be a reliable anatomical landmark to set both the frontal and axial alignment of the femoral component in medial UKA.

**Methods:**

73 patients undergoing medial UKA had pre-operative CT imaging to generate 3D models. Those with osteophytes that impaired visualisation of the condylar walls were excluded. 28 patients were included in the study. The ideal KA was determined using the cylindrical axis in the frontal and axial plane. Simulations using the medial wall of the medial condyle (MWMC) and the lateral wall of the medial condyle (LWMC) were performed to set the frontal alignment. To set the axial alignment, the MWMC, LWMC, medial wall of the lateral condyle (MWLC), and medial diagonal line (MDL) anatomical landmarks were investigated. Differences between the ideal measured KA values and values obtained using landmarks were investigated.

**Results:**

Use of the MWMC let to similar frontal alignment compared to the ideal KA (2.9° valgus vs 3.4° valgus, *p* = 0.371) with 46.4% (13/28) of measurements being $\le$ 1.0° different from the ideal KA and only 1 simulation with greater than 4.0° difference. Use of the MWMC led to very similar axial alignments compared to the ideal KA (0.5° internal vs 0.0°, *p* = 0.960) with 75.0% (21/28) of measurements being $\le$ 1.0^o^ different from the ideal KA, and a maximum difference of 3.0°. Use of the MWLC and MDL was associated with significant statistical differences when compared to the ideal KA (*p* < 0.001 for both).

**Conclusions:**

The native orientation of the medial condylar wall seems to be a reliable anatomical landmark for aligning the femoral component in medial KA UKA in both the axial plane and frontal planes. Other assessed landmarks were shown to not be reliable. Clinical and radiographic assessments of the reliability of using the MWMC to set the frontal and axial orientation of the femoral component when performing a medial KA UKA are needed.

## Introduction

Conventionally, unicompartmental knee arthroplasty (UKA) implant alignment has been standardised to be parallel to the femoral and tibial mechanical axis for the femoral and tibial components, respectively [7, 14]. However, implants may be aligned using an alternative technique known as kinematic alignment (KA). In KA, the implant is aligned to be perpendicular to the cylindrical axis in both the frontal and axial planes [12]. As such, it aims to maintain more physiological biomechanics and offers a personalised approach to UKA where patients’ individual anatomy, laxity, and kinematics are more considered [2]. The technique aims to restore the patient’s articular surface orientation and height as well as bone loading [10]. A kinematically aligned implant in medial UKA is likely to offer advantageous knee kinematics, and such implantations have been shown to be safe, with a good efficacy [9].

A common issue during medial KA UKA surgery is the inability to be able to identify the required kinematic axes when performing mini-invasive sub-quadricipital approach. Therefore, alternative methods with the utilisation of anatomical landmarks would benefit the reproducibility of KA UKA, if proved reliable. When kinematically aligning the femoral component during UKA, the component requires precise alignment in both the frontal and axial planes, with the goal to end up perpendicular to the cylindrical axis in both planes. Alignment in the frontal plane involves altering the varus/valgus orientation, whereas axial alignment involves altering the external/internal rotation.

This study aims to assess if the orientation of these condylar walls may be suitable for use as an anatomical landmark to kinematically align the femoral component. This in-silico study aimed to answer the following questions:

1. Are the medial and lateral walls of the medial condyle suitable as anatomical landmarks to set the varus/valgus alignment of the femoral component in the frontal plane in medial UKA?
2. Are the medial and lateral walls of the medial condyle as well as alternative anatomical landmarks suitable to set the internal/external rotation in the axial plane of the femoral component in medial UKA?

It was hypothesised that the medial condylar wall would prove to be a reliable anatomical landmark to set both the frontal and axial alignments of the femoral component in medial UKA.

## Materials and methods

**Fig. 1:**
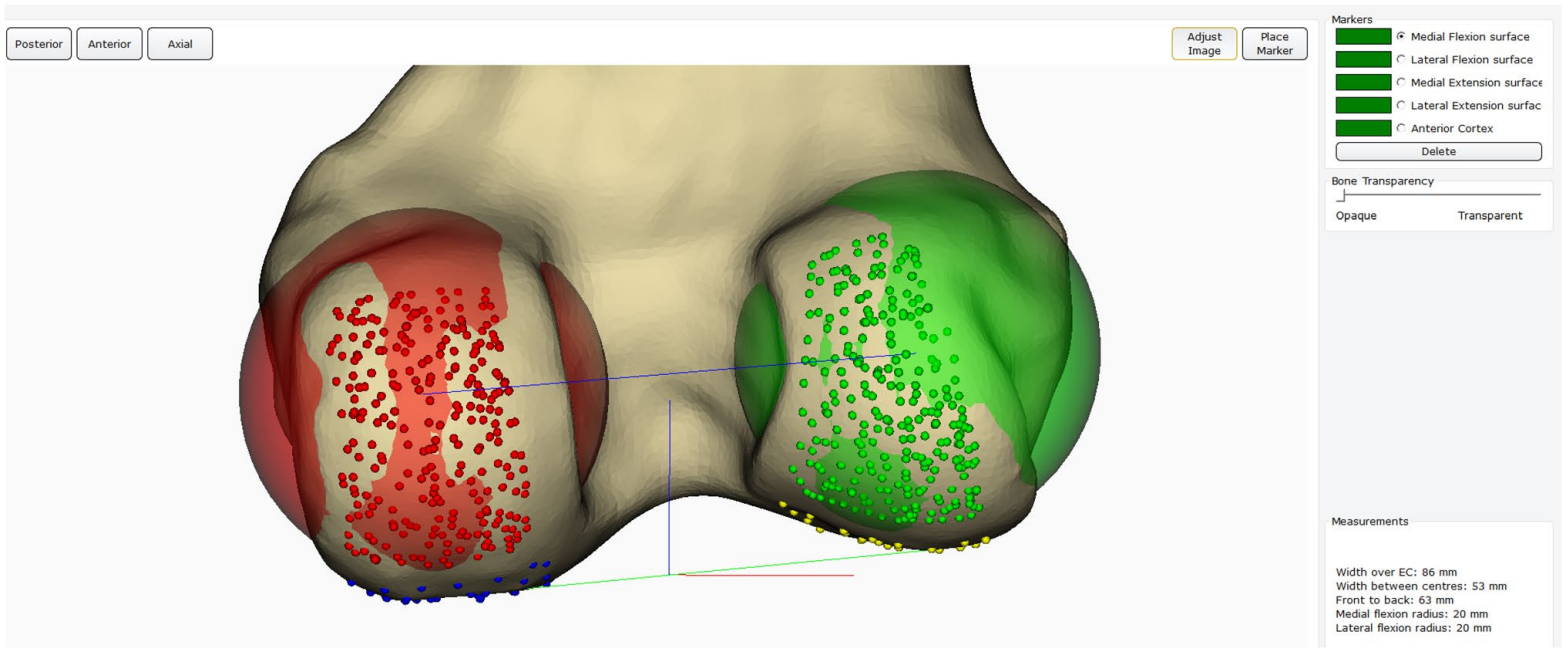
Example of landmarking of the medial and lateral flexion surfaces to allow computation of the cylindrical axis. Horizontal line passing through the centres of the medial and lateral condyles represents the computed cylindrical axis based on this landmarking

To address these aforementioned questions, this computational study was carried out. 73 patients undergoing medial UKA had pre-operative CT scans which were performed using a protocol based on the Imperial Knee Protocol [6]. A Siemens Somaton Definition AS + 128 slice scanner (Siemens Medical Solutions, Erlangen, Germany) was used, with the X-ray source set to 100 kilovolts and 100 milliamp seconds and a collimation width of 0.6 mm. Scans were then reconstructed with 1 mm spaced-slices at the knee and output as DICOM data. The CT slices were 512 × 512 voxels with a field of view that covered both legs and hips. Although this can vary based on the size of the patient, it was typically 350 mm which yielded a voxel size of around 0.68 mm × 0.68 mm x 1 mm at the knees. The data were then imported into Materialise Mimics® software (Materialize, Belgium) and segmented to generate 3D models of their femur. These models were loaded into in-house planning software that allowed the user to simulate alignments of the femoral component of an Oxford® mobile-bearing medial UKA implant (Zimmer-Biomet, Warsaw, Indiana, USA). Knees with osteophytes that impaired the visualisation of condylar walls were excluded. Following this, 28 patients were included in this study. Each knee then underwent landmarking of the flexion and extension surfaces to allow the software to generate the cylindrical axis (Fig. 1). Alignments were measured to the nearest 0.5°.

**Fig. 2:**
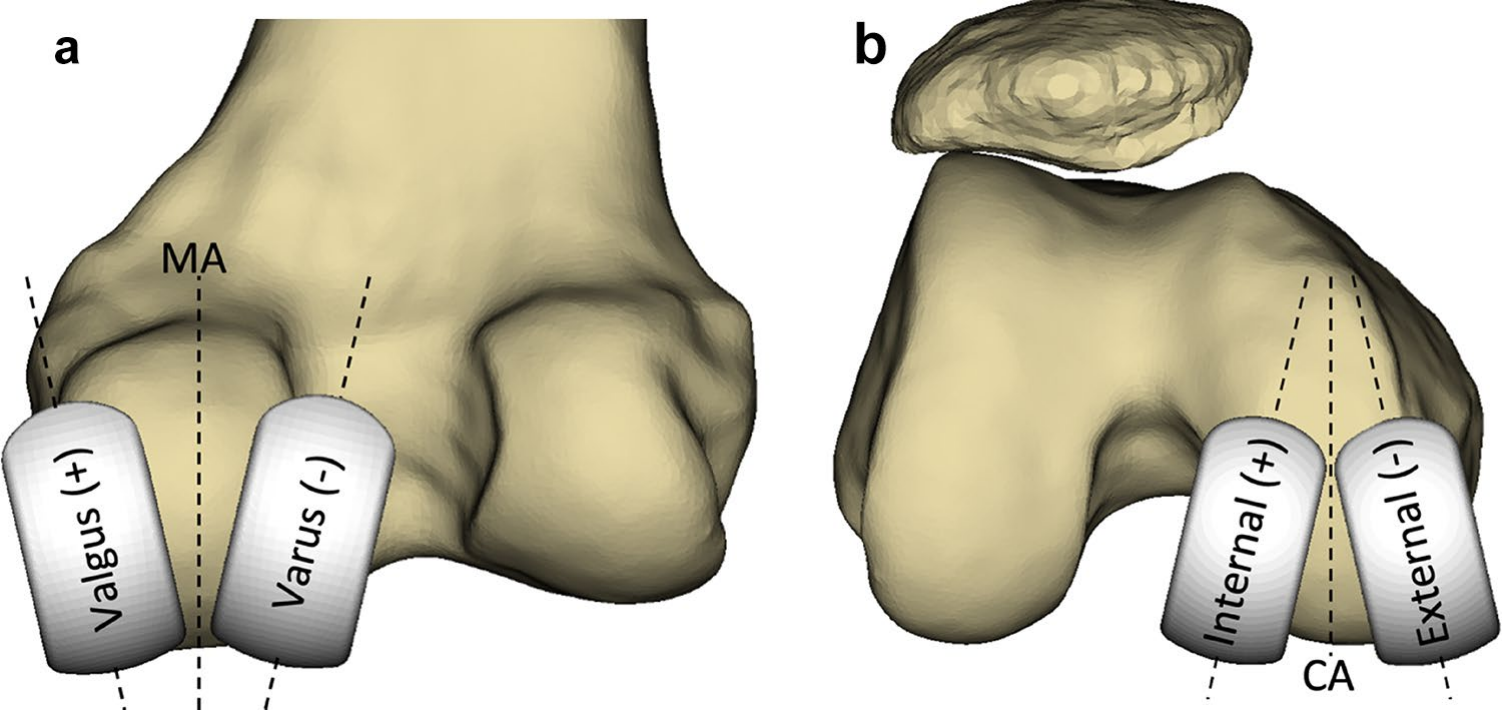
**A** Effect of changing varus/valgus orientation of the femoral component in the frontal plane. Dotted line in centre represents the mechanical axis (MA) of the femur which varus/valgus values were measured relative to. **B** Effect of changing internal/external rotation of the femoral component in the axial plane. Dotted line in centre represents a line perpendicular to the cylindrical axis (CA) in the axial plane

The varus/valgus orientation of the femoral component was altered to align the implant in the frontal plane (Fig. 2A). Varus values were considered to be negative with valgus values considered positive. The internal/external rotation of the femoral implant was altered to align the implant in the axial plane (Fig. 2B). Externally rotated implants were considered to be negative with internally rotated components considered positive. Values were measured relative to the femoral mechanical axis in the frontal plane and relative to the cylindrical axis in the axial plane.

To assess the use of anatomical landmarks, a perfect KA was simulated to compare against. The varus/valgus alignment of the femoral implant in a perfect KA in the frontal plane was determined using the computed cylindrical axis, so that the implant was aligned perpendicular to this axis.

**Fig. 3:**
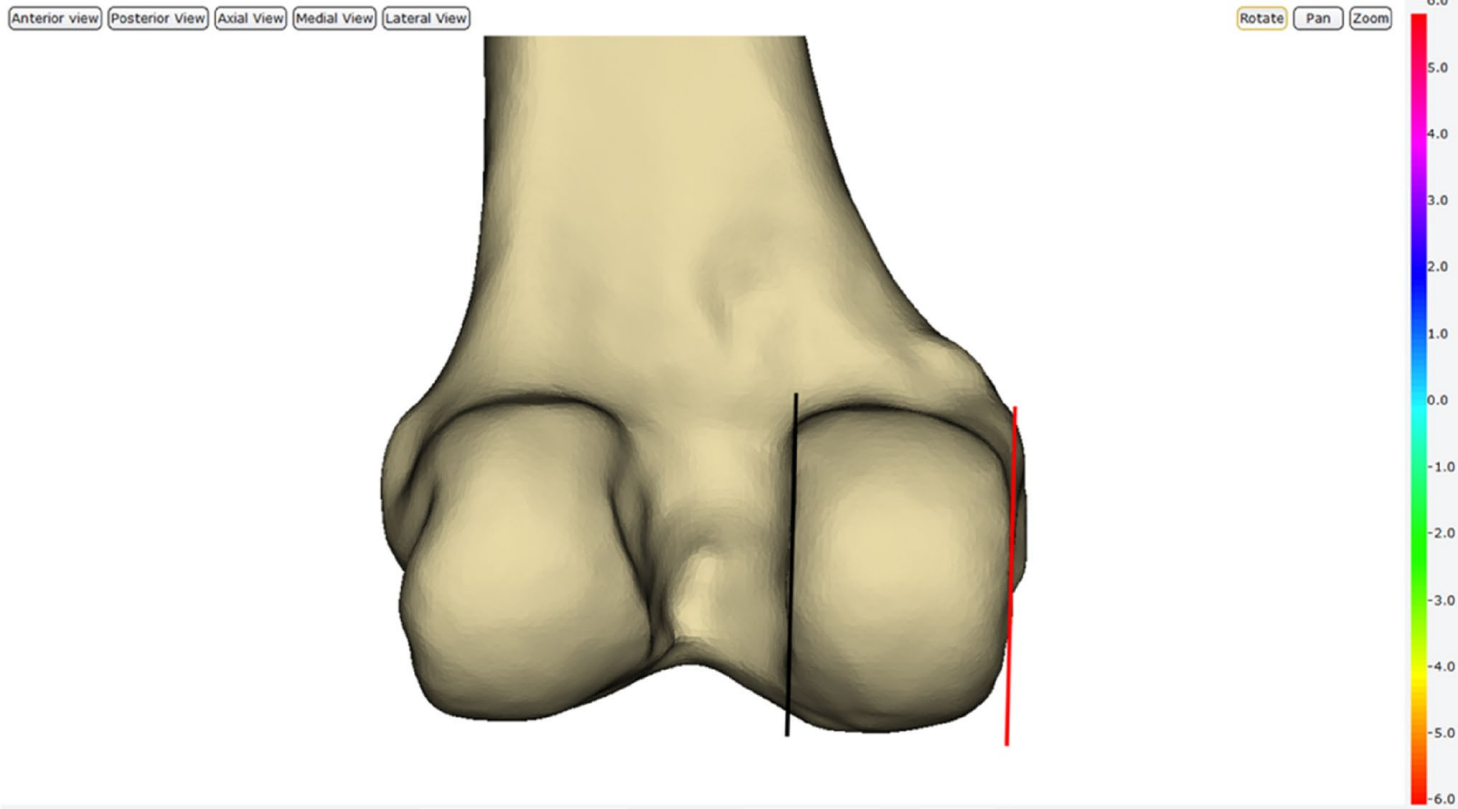
Model showing the position of alignments for setting the varus/valgus orientation in the frontal plane. Red line represents the medial wall of the medial condyle (MWMC) in which the implant was aligned to be parallel to. Black line represents the lateral wall of the medial condyle (LWMC) in which the implant was aligned parallel to

The anatomical landmarks that were assessed for setting the varus/valgus orientation in the frontal plane were the medial wall of the medial condyle (MWMC) and the lateral wall of the medial condyle (LWMC). To assess the use of the MWMC as a landmark, femoral implants were manually aligned, so that their varus/valgus orientation was parallel to that of the MWMC (Fig. 3). Use of the LWMC as an anatomical landmark was assessed using an identical method except the varus/valgus alignment was set, so that the implant was parallel to the lateral wall of the medial condyle.

To determine the use of anatomical landmarks for setting the internal/external alignment of the femoral component in the axial plane, the alignment for an ideal KA was required. This was determined using the computed cylindrical axis, so that the implant was aligned perpendicular to this axis.

**Fig. 4:**
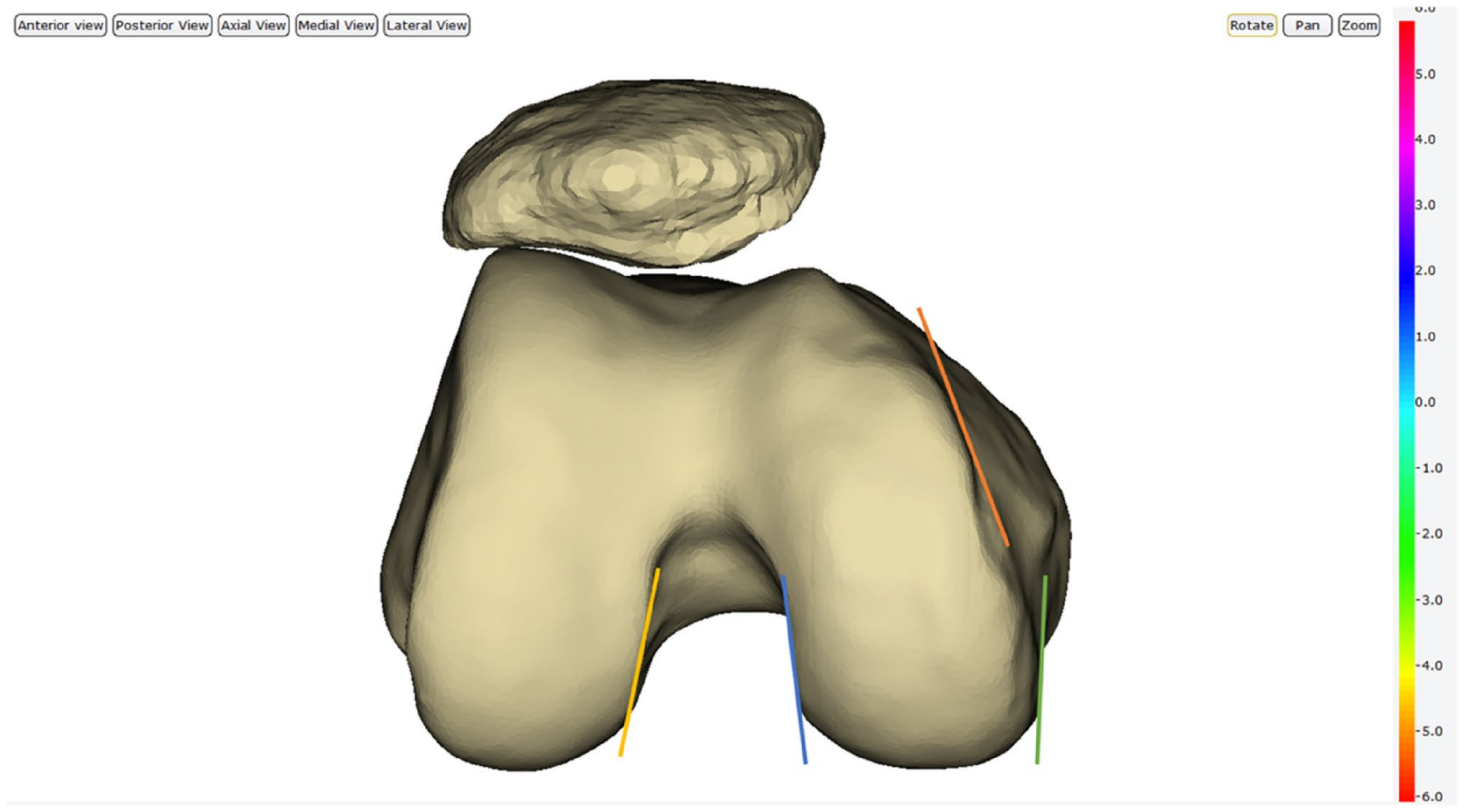
Model showing the anatomical landmarks for setting the internal/external rotation of the implant in the axial plane. Different coloured lines represent different landmarks that were used to align the implant parallel to. Green line represents the medial wall of the medial condyle (MWMC), blue line represents the lateral wall of the medial condyle (LWMC), yellow line represents medial wall of the lateral condyle (MWLC), and orange line represents the medial diagonal line (MDL)

The anatomical landmarks that were then assessed for setting the internal/external rotation in the axial plane were the MWMC, LWMC, medial wall of the lateral condyle (MWLC), and the medial diagonal line (MDL). To assess the use of the MWMC, the internal/external rotation of the implant was adjusted, so that the implant was aligned parallel to the medial wall. The same process was used to investigate the use of the LWMC as a landmark. As shown in Fig. 4, when using the MWLC as an anatomical landmark, the internal/external rotation of the implant was adjusted, so that the implant was parallel to this wall. The anatomical landmark which was assigned as the MDL was also investigated. This line corresponded to the apparent diagonal line formed by the medial condyle. The internal/external rotation of the implant was then adjusted to be parallel to this so-called MDL.

As images were anonymised, no institutional review board approval was needed.

### Statistical analysis

Differences between the simulated ideal KA values measured using the cylindrical axis were compared to the measured varus/valgus values for both medial and lateral wall anatomical landmarks in the frontal plane to identify any statistically significant differences. Differences between the ideal KA values and the measured internal/external rotation values in the axial plane for the medial and lateral wall anatomical landmarks were compared to identify any significant differences. Additionally, the internal/external rotation values measured for the MWLC and the MDL landmarks were compared to the ideal KA in the axial orientation to identify significant differences. Two-tailed Student’s *t* tests were used to test for any statistically significant differences with *p* values < 0.05 considered to be significant.

The degree difference between measured ideal KA values and the values obtained using anatomical landmarks in both the frontal and axial planes were also calculated to determine the percentage of results that had a difference of ≤ 1.0°, > 1.0°, > 2.0°, > 3.0°, > 4.0°, and > 5.0^o^ compared to the ideal KA value.

All data were presented as means, standard deviation (SD), and minimum and maximum values which were suitable due to the normal distribution of data. Additionally, all values were measured to 1 decimal place. As well as calculating the mean alignment when using anatomical landmarks in each plane, the absolute values were used to calculate the mean degrees that the implant was deviated away from the ideal KA when using different anatomical landmarks. The standard deviation, and minimum and maximum values were also calculated for this using absolute values.

To assess intra-observer reliability of measurements, 10 patients were randomly selected and measurements for each landmark were repeated with blinding from the originally measured values. Inter-observer reliability of measurements was also assessed using an external researcher that had not taken part in the original alignments. They performed alignments of the same 10 patients with blinding from original values. The reliability of our measurements was assessed using an intraclass correlation coefficient (ICC) two-way mixed-effects model which reported average measures and the 95% confidence interval in brackets. Using the guidelines recommended by Cicchetti and Sparrow [1], the ICC for intra-rater reliability was excellent for the MWMC and LWMC in the frontal plane. This is seen by an average measures ICC value of 0.954 (0.824–0.988) and 0.969 (0.770–0.993), respectively. The ICCs for intra-rater reliability were also excellent for the MWLC, LWMC, MWLC, and MDL in the axial plane. This is seen by average measures ICC values of 0.951 (0.800–0.988), 0.956 (0.825–0.989), 0.987 (0.916–0.997), and 0.946 (0.786–0.987), respectively. For inter-rater reliability, the ICC values were excellent for both the MWLC and LWMC in the frontal plane as seen by ICC values of 0.977 (0.912–0.994) and 0.946 (0.771–0.987), respectively. The ICC values for inter-rater reliability were also found to be excellent for the MWMC, LWMC, MWLC, and MDL. This is seen by an average measures ICC of 0.944 (0.776–0.986) and 0.944 (0.786–0.986) for the MWMC and LWMC, respectively, and 0.969 (0.790–0.993) and 0.939 (0.770–0.985) for the MWLC and MDL, respectively.

## Results

### Setting the varus/valgus alignment in the frontal plane using anatomical landmarks

The mean varus/valgus alignment of an ideal KA implant was found to be 3.4° valgus (SD 1.6°, from 0.0°–7.5° valgus).

The mean varus/valgus alignment when aligning the implant parallel to the MWMC was 2.9° valgus (SD 2.4°, from − 1.5° varus to 7.5° valgus), with no significant statistical difference with the ideal KA simulation (*p* = 0.371). Compared to the measured varus/valgus alignment in the ideal KA simulation, using the MWMC generated 46.4% (13/28) of results within or equal to 1.0° varus/valgus of the ideal KA values, and 3.6% (1/28) of simulations with greater than 4.0° difference (Table 1). When using absolute values, use of the MWMC led to a mean implant frontal orientation of 1.8° away from the ideal KA value (SD 1.4°, from 0.0° to 6.5°).

**Table 1:** Percentage of patients (%) with a varus/valgus alignment when using the MWMC or LWMC as a landmark that is $\le$ 1.0°, > 1.0°, > 2.0°, > 3.0°, > 4.0°, and > 5.0^o^ of the originally measured kinematically aligned value

	Medial wall of medial condyle	Lateral wall of medial condyle
% of results $\le$ 1.0° of KA value	46.4	39.3
% of results > 1.0° of KA value	53.6	60.7
% of results > 2.0° of KA value	32.1	39.3
% of results > 3.0° of KA value	14.3	25.0
% of results > 4.0° of KA value	3.6	17.9
% of results > 5.0° of KA value	3.6	10.7

The mean varus/valgus alignment when aligning the implant parallel to the LWMC was 2.6° valgus (S.D 3.7°, from − 5.0° varus to 7.5° valgus), with no significant statistical difference compared to the ideal KA simulation (*p* = 0.286). When using the LWMC for alignment, 39.3% (11/28) of results were within or equal to 1.0° varus/valgus of the ideal KA value, and 17.9% (5/28) had a greater than 4.0° difference. Use of the LWMC was led to a mean implant frontal orientation that was 2.4° away from the ideal KA value (SD 2.2°, from 0.0° to 8.0°).

### Setting the internal/external rotation in the axial plane using anatomical landmarks

In the axial plane, an ideal KA implant is perpendicular the cylindrical axis. Therefore, the ideal KA was set to be 0.0° internal/external rotation, with subsequent alignments being measured relative to this.

The mean internal/external rotation when using the MWMC was 0.5° internal (SD 1.4°, from − 2.0° external to 3.0° internal) with no significant statistical difference with the ideal KA simulation (*p* = 0.960). 75.0% (21/28) of values was within or equal to 1.0° of the ideal KA alignment with 0.0% (0/28) of alignments being greater than 3.0° away from the ideal KA (Table 2). When using absolute values, use of the MWMC led to a mean axial rotation that was 1.2° away from the ideal KA value (SD 0.9° from 0.0° to 3.0°).

**Table 2:** Percentage of patients (%) with an internal/external rotation when using the medial wall of the medial condyle, lateral wall of the medial condyle, medial wall of the lateral condyle, and medial diagonal line as a landmark that is $\le$ 1.0°, > 1.0°, > 2.0°, > 3.0°, > 4.0°, and > 5.0^o^ of the originally measured kinematically aligned value

	Medial wall of medial condyle	Lateral wall of medial condyle	Medial wall of lateral condyle	Medial diagonal line
% of results $\le$ 1.0° of KA value	75.0	32.1	0.0	0.0
% of results > 1.0° of KA value	25.0	67.9	100.0	100.0
% of results > 2.0° of KA value	17.9	60.7	100.0	100.0
% of results > 3.0° of KA value	0.0	39.3	100.0	100.0
% of results > 4.0° of KA value	0.0	35.7	92.9	100.0
% of results > 5.0° of KA value	0.0	25.0	89.3	100.0

Use of the LWMC led to a mean axial alignment of 1.3° internal (SD 4.6°, from − 5.5° external to 11.0° internal), with no significant statistical difference with the ideal axial KA (*p* = 0.345). When using the LWMC, 32.1% (9/28) of simulations were within or equal to 1.0° of the ideal KA simulation, with 25.0% (7/28) of simulation greater than 5.0° away from the ideal KA simulation. Use of the LWMC led to a mean alignment that was 3.7° rotated away from the ideal KA (SD 3.0°, from 0.0° to 11.0°).

The mean internal external rotation when aligning the implant parallel to the MWLC was 10.9° internal (SD 7.0° from -6.0° external to 22.0° internal) with a significant statistical difference with the ideal KA simulation (*p* < 0.001). When using the MWLC, 0.0% (0/28) of values measured were within or equal to 1.0° of the ideal KA simulation with 89.3% (25/28) of simulations greater than 5.0° of the ideal KA simulation. The mean alignment when using the MWLC was 11.8° from the ideal KA (SD 5.4°, from 3.5° to 22.0°).

The mean axial alignment when using the MDL was 18.2° external (SD 2.9°, from − 21.5° external to − 10.0° external) with a significant statistical difference with the ideal KA simulation (*p* < 0.001). Additionally, 100.0% (28/28) of all values were greater than 5.0° of the measured ideal KA value. Use of the MDL led to a mean alignment that was 18.2° away from the ideal KA (SD 2.8°, from 10.0° to 21.5°).

## Discussion

The most important finding of the present study was that the native orientation of the MWMC seems to be a reliable anatomical landmark for setting the axial (internal/external rotation) and frontal (varus/valgus) orientation of a kinematically aligned femoral implant. Precise KA UKA implantation implies that implants are aligned perpendicular to the cylindrical axis in the frontal and axial planes [3–5]. However, when performing mini-invasive (sub-quadricipital) medial UKA, only the medial compartment of the knee is visible to the surgeon which makes identification of the cylindrical axis challenging [15]. This study was initiated to assess the value of the orientation (or anatomy) of the medial femoral condyle to locate (indirectly) the cylindrical axis and improve the reproducibility of medial KA UKA.

**Fig. 5:**
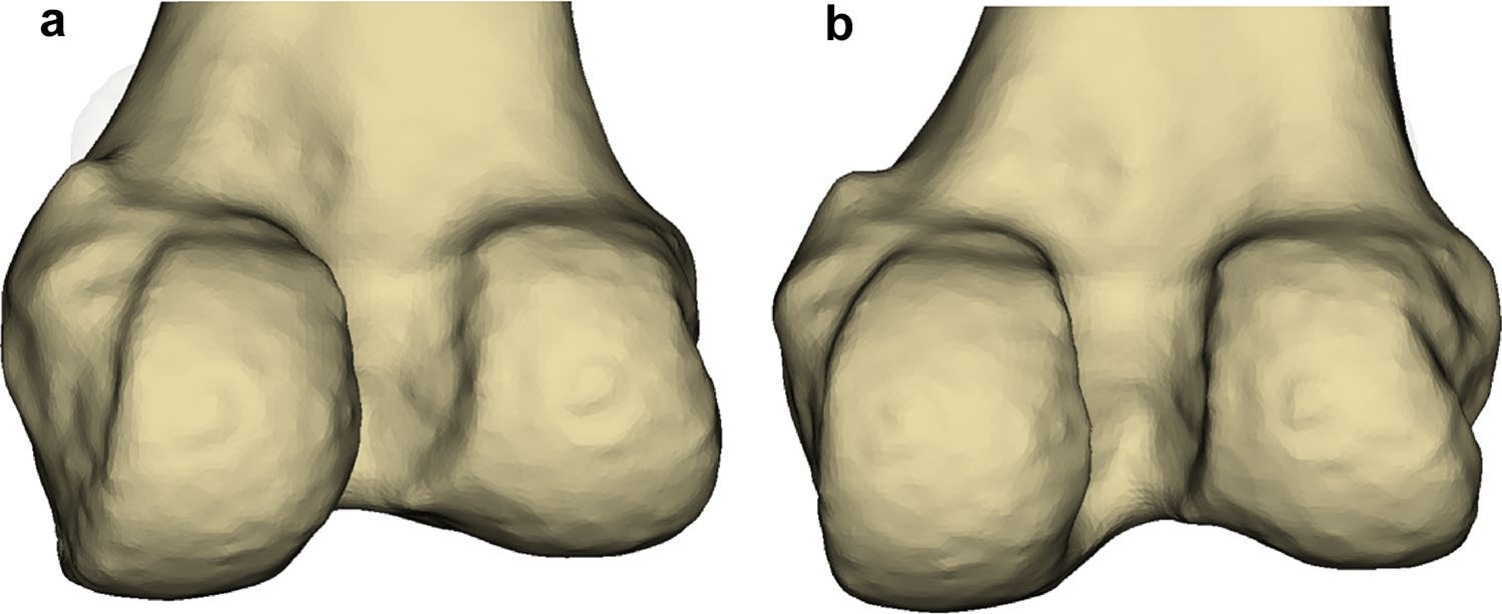
Model of the patient with a 6.5^o^ difference between MWMC and ideal KA alignment. **A** Medial view of the MWMC and **B** posterior view showing the medial condyle anatomy

When using the MWMC to set the varus/valgus alignment, only one of the 28 simulations had a difference in varus/valgus alignment of greater than 5.0° compared to the ideal KA. This makes the MWMC a suitable anatomical landmark to set the frontal orientation of a medial KA UKA, for most patients. However, one simulation led to a 6.5° difference between ideal and MWMC simulation. As seen in Fig. 5, this patient had no significant osteophytes, suggesting that this large difference was simply due to the shape of the patient’s native medial wall. It is likely that a large mismatch between the frontal orientation of the femoral and tibial implants could lead to poor implant interaction in the knee extension position, with risk of mobile liner dislocation [13]. However, this risk would probably be low, because the mobile-bearing and single radius design of the Oxford™ UKA makes it a forgiving implant regarding the risk of poor implants’ interaction (or interplay) [8]. To lower this risk, the MWMC could be used in conjunction with other methods to help improve the reliability of frontal KA positioning of the femoral UKA implant, mainly in situations where the surgeon has difficulty in assessing the native frontal orientation of the MWMC (e.g., improper cleaning of condylar osteophytes). For example, pre-operative planning to define the varus/valgus orientation of the distal femoral joint line relative to the femoral mechanical axis, which indicates the frontal orientation of the cylindrical axis, could improve the reliability of frontal KA positioning of the femoral UKA implant [3–5]. This corresponds to the practice of the last author (CR), using in conjunction 1) the orientation of the MWMC that is displayed by a K-wire inserted along it (after cleaning medial osteophytes and by making sure that medial soft tissues are not touching the K-wire, therefore altering its orientation), and 2) the measured varus-valgus orientation of the distal femoral joint line relative to the femoral mechanical axis [11].

When using the MWMC, approximately three quarters of simulations were almost perfectly axially aligned (within or equal to 1.0°) and none (0/28) had more than 3.0° difference compared to the ideal KA simulation. This makes the MWMC a reliable anatomical landmark to set the axial KA orientation of the femoral UKA implant. Other anatomical landmarks (LWMC, MWLC, and MDL) assessed in this study for setting the axial KA orientation of a medial UKA proved to be unsuitable with high rates of large deviations (> 5.0°) from the ideal KA simulation.

The findings from this study may contribute to improving the precision and reliability of UKA KA implantation. However, it is important to acknowledge a few limitations of our methods which may affect the generalisation of our results. First, the models used in this study were all from knee osteoarthritic patients that were undergoing UKA. As such, this meant that multiple knees had osteophytes which obscured visualisation of the true condylar walls making alignment of implants challenging in some patients. This was minimised by excluding knees with osteophytes that impaired the visualisation of condylar walls. It is also worth noting that during surgery, osteophytes can easily be identified and removed due to their altered structure compared to native bone. However, due to the computational nature of this study, this was not possible. Second, the small sample size of 28 knees used in this study may not be large enough to give a reliable overview of knee anatomy for those undergoing UKA. Third, the 3D models did not include cartilage which might have altered the orientation of the condylar walls, although this is likely to have had a negligible effect. Finally, since this is an in-silico, the findings from this study cannot be extrapolated to clinical practice.

## Conclusion

This study has shown that the native orientation of the medial condylar wall may potentially serve for aligning the femoral component in medial KA UKA in both the axial and frontal planes. Other assessed landmarks were shown to not be reliable. Clinical and radiographic assessments of the reliability of using the MWMC to set the frontal and axial orientation of the femoral component when performing a medial KA UKA are needed.
